# Microdeletions in 1q21 and 8q12.1 depict two additional molecular subgroups of Silver-Russell syndrome like phenotypes

**DOI:** 10.1186/s13039-022-00596-z

**Published:** 2022-05-13

**Authors:** Naomi Baba, Anna Lengyel, Eva Pinti, Elzem Yapici, Isolde Schreyer, Thomas Liehr, György Fekete, Thomas Eggermann

**Affiliations:** 1 Institute of Human Genetics, University of Jena, Jena, Germany; 2 Praxis Für Humangenetik, Zentrum Für Ambulante Medizin, Jena, Germany; 3 2Nd Department of Pediatrics, Semmelweis University Budapest, Budapest, Hungary; 4 Institute of Human Genetics, Medical Faculty, RWTH Aachen University, Pauwelsstr. 30, 52074 Aachen, Germany

**Keywords:** Silver-Russell syndrome (SRS), *PLAG1*, 1q21.q21.2 deletion, Molecular karyotyping

## Abstract

**Background:**

Silver-Russell syndrome (SRS) is a genetic disorder characterized by intrauterine and postnatal growth restriction, relative macrocephaly at birth, body asymmetry and typical facial features. Clinical and molecular heterogeneity is described in SRS. Common causes are loss of methylation of the imprinting center 1 in 11p15 and maternal uniparental disomy of chromosome 7. Other genetic alterations include disturbances of imprinted regions in 14q32, 7q32 and 11p15 as well as submicroscopic deletions and duplications. Single nucleotide variants in genes like *IGF2, HMGA2, PLAG1, CDKN1C* have also been identified in patients with SRS phenotypes. However, routine molecular diagnostics usually focus on 11p15 and chromosome 7, while less frequent causes are not systematically addressed.

**Results:**

Here we report two patients with SRS features in which molecular karyotyping revealed microdeletions in 1q21 and 8q12.1 respectively. In a 3.5-year-old girl with postnatal growth restriction, feeding difficulties, relative macrocephaly and distinct SRS features a 2 Mb deletion in 1q21.1q21.2 was identified. Our second case is a 1.5-year-old boy with intrauterine and postnatal growth restriction, feeding difficulties and distinct facial features with a 77 kb deletion in 8q12.1 affecting *PLAG1* as the only protein-encoding gene with known function.

**Conclusions:**

The 1q21 region has not yet been assigned as an SRS region, although six patients with the same deletion and SRS features including relative macrocephaly have been described before. This new case adds to the evidence that distal 1q21 should be annotated as an SRS candidate region. The *PLAGL1* alteration is the smallest deletion in 8q12.1 ever reported in a patient with SRS phenotype and it finally confirms that *PLAG1* is the SRS causing gene in 8q12.1. To increase the diagnostic yield in patients with suspected SRS, we recommend both molecular karyotyping and next generation sequencing-based approaches.

## Background

Silver-Russell syndrome (SRS) is a congenital disorder, mainly characterized by severe intrauterine and postnatal growth restriction, relative macrocephaly at birth, body asymmetry and a typical facial gestalt (for review: [1]). Due to a lack of specificity of these symptoms, and clinical heterogeneity, SRS is often discussed being a differential diagnosis and is genetically tested in patients with growth retardation. Consequently, the detection rate for the currently known molecular disturbances of SRS is only 10–20% (averaged detection rate in different diagnostic centers (unpublished data, [2]), whereas in clinically well characterized cohorts of SRS patients it reaches nearly 70%.

The major diagnostically accessible alterations in SRS comprise: detecting (i) loss of methylation (LOM) in imprinting center 1 (IC1, H19/IGF2:IG-DMR in 11p15—accounting for up to 50% of cases), (ii) maternal uniparental disomy of chromosome 7 (upd[7]mat—in up to 10% of the cases), (iii) disturbances of imprinted regions in 14q32, 7q32 and 11p15, and/or (iv) submicroscopic deletions and duplications (CNVs, copy number variations) (for review: [3]). Additionally, (v) single nucleotide variants (SNVs) in different genes have been identified. These mutations are either associated with the SRS or SRS like phenotypes (*IGF2, HMGA2, PLAG1, CDKN1C*), or with differential diagnoses of SRS (e.g. [4–6]).

Whereas procedures to uncover molecular alterations affecting 11p15 and chromosome 7 are already implemented in routine molecular diagnostics of SRS [1], analysis of further genomic regions have been suggested as second line testing [1]. However, they are not systematically addressed in the general diagnostic workup of SRS. In fact, the analysis of both microarray 7 molecular karyotyping analysis and next generation sequencing (NGS)-based approaches significantly increases the detection rate in this heterogeneous clinical cohort [1, 3, 5, 7].

In addition to the major molecular SRS subgroups in 11p15, chromosome 7 and 14q32, genomic alterations within 1q21 and 8q12.1 have recently come into focus as SRS causing regions (for review: [3]).

**Fig. 1:**
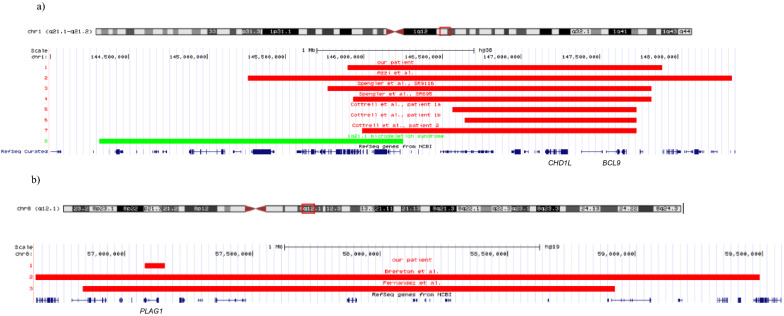
UCSC custom track illustrating the extents of the 1q21 and 8q12 deletions. **a** The region of interest on chromosome 1 is indicated by the red box. Our patient (red bar 1) carries a 2 Mb deletion in 1q21.1q21.2 (arr(GRCh37) 1q21.1q21.2(145,895,747_147,897,962) × 1). To compare the extent of our patient’s deletion to patients from the literature (Azzi et al^4^, Spengler et al.[8], Cottrell et al. [10] only the SRS candidate genes discussed by [10] are shown. The green bar displays the well known 1q12.2 microdeletion syndrome**.** **b** The region of interest on chromosome 8 is indicated by the red box. Our patient (red bar 1) carries a 77 kb deletion in 8q12.1 affecting the *PLAG1* gene (arr[GRCh37] 8q12.1(57,079,399_57,155,945) × 1). Note the larger deletions in patients from the literature [12, 13]. (Among the numerous genes affected in the reported cases, only the *PLAG1* gene is indicated)

The first cases of microscopic deletions in 1q21 have been published by Spengler et al. [8] (Table 1a, Fig. 1a), and meanwhile four further cases with such alterations have been identified by molecular karyotyping in suspected SRS patients [9, 10]. Furthermore, genomic alterations of the *PLAG1* gene in 8q12.1 have recently been identified in patients with an SRS phenotype, including both SNVs [4–6, 11] and CNVs [12, 13] (Table 1b, Fig. 1b); *PLAG1* has therefore been suggested as SRS candidate gene. Accordingly, a fourth OMIM entry has been assigned for SRS as SRS4 (OMIM#618,907). These patients have either been identified by NGS-based approaches (whole exome sequencing, WES; targeted panel sequencing) ([4–6, 11]) or molecular karyotyping [12, 13]. In fact, the first patient with an 8q12.1 deletion has been identified by banding cytogenetics in 1994 by Schinzel and colleagues [14].

**Table 1:** Comparison of the newly identified patients with reported patients with deletions in 1q21.1q21.2 and 8q12.1 of similar sizes. In fact, in several public databases (DECIPHER, ClinVar), further cases with deletions affecting these regions are documented, but none of them exhibited alterations of similar size. Nearly all patients have been referred for molecular karyotyping due to clinical features of SRS, but only the NHS criteria are shown here

	Symptoms	Our case	[9]	[8] SR9116	[8] SR5695	[10] patient 1a	[10] patient 1b	[10] patient 2	Frequency
*a Information on 1q21.1q21.2 cases*									
Variant	Affected 1q21.1q21.2 region (GRCh37)	145,895,747–147,897,962	145,261,451–148,343,412	145,770,626–147,831,171	145,932,455–147,831,171	146,564,742–147,735,011	146,641,600–147,735,011	145,987,155–147,735,011	
	Size of the deletion	2 Mb	3 Mb	2 Mb	1.9 Mb	1.17 Mb	1.09 Mb	1.74 Mb	
	Parental origin	de novo	de novo	maternal	not maternal	maternal	maternal	de novo	
NH-CSS criteria	SGA/IUGR	0	0	1	1	NA	NA	NA	50.0% (2/4)
	Height at 2 years (< -2 SD)	0	1	1	1	1	NA	1	83.3% (5/6)
	Relative macrocephaly	0	0	NA	0	NA	NA	1	25.0% (1/4)
	Feeding difficulties	1	1	NA	1	1	1	1	100.0% (6/6)
	body asymmetry	0	1	0	0	NA	NA	NA	25.0% (1/4)
	Protruding forehead	0	1	0	1	NA	NA	NA	50.0% (2/4)
	NH-CSS	1/6	4/6	2/4	4/6	2/2	1/1	3/3	

	Symptoms	Our case	[13] Patient 1	[13] Patient 2	[12]	[14]	SNV cases, reviewed by [13]	Frequency
*b Molecular and clinical information on 8q12.1 patients*								
Variant	Affected 8p21.1 region (GRCh37)	57,079,399–57,155,945	56,609,388–59,488,289		56,834,331–58,921,491	46,XX,del[8](q11q12)	n = 8 (cases for which information was available)	
	Size of the deletion	77 kb	2.9 Mb	2.1 Mb	NA			
	Origin	de novo	Maternal	de novo	de novo			
NH-CSS criteria	SGA/IUGR	0	0	1	1	1	6 (6)	81.8% (9/11)
	Height at 2 years (< -2 SD)	0	NA	0	1	1	5 (5)	88.9% (8/9)
	Relative macrocephaly	0	0	1	1	0	2 (5)	44.4% (4/9)
	Feeding difficulties	1	1	1	1	NA	6 (6)	100.0% (10/10)
	Body asymmetry	0	0	0	0	0	0 (7)	0.0% (0/12)
	Protruding forehead	1	1	1	0	NA	5 (6)	80.0% (8/10)
	NH-CSS	2/6	2/5	4/6	4/6	2/4	Score ≥ 4/6: 4 (5)	60.0% (6/10)

IUGR = intrauterine growth retardation; SD = standard deviation; SGA = small for gestational age; NA = not assessed, NH-CSS = Netchine-Harbison clinical scoring system

Based on the identification of two further cases with SRS features and microdeletions in 1q21 and 8q12.1, respectively, and data from the literature, we show that these molecular alterations present two further molecular subgroups of the SRS phenotype. We suggest the adjustment of the molecular diagnostic workup regarding these new molecular findings in SRS patients, and the comprehensive application of available testing methods.

## Results

### Case 1

A de-novo 2 Mb deletion in 1q21.q21.2 was identified in a 3.5 year-old girl. She is the second of three children of healthy, consanguineous Syrian parents.

She was born at 38 + 4 gestational weeks with reduced weight (2750 g, z − .26) and length (47 cm, z − 1.7) and a head circumference of 32 cm (z − 0.91).

During pregnancy, the mother suffered from gestational diabetes, which was treated with modified diet. Intrauterine growth retardation (IUGR) was not reported, however, fetal hydronephrosis was diagnosed prenatally. Postnatally, vesicoureteral reflux led to recurrent episodes of pyelonephritis eventually making a reimplantation of both ureters necessary. In the postnatal period, the patient experienced feeding difficulties and a consecutive failure to thrive. Additional sip feed nutrition was started at the age of 2 years. The motoric and linguistic developmental milestones were reached within the normal range of time.

**Fig. 2:**
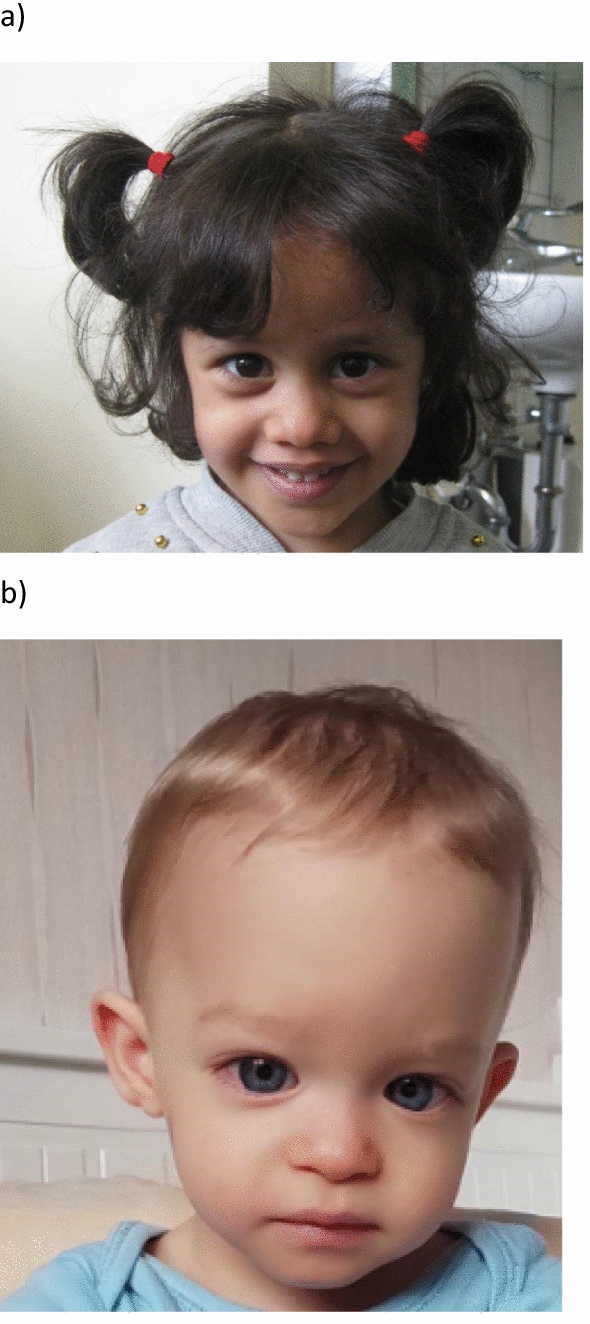
**a** A 3.5-year-old girl with a 2 Mb deletion 1q21.1q21.2. Note the triangular face and slightly asymmetrical palpebral fissures. **b** A 1.5-year-old boy with a 77 kb deletion 8q12.1. Note the high forehead, epicanthal folds, low set ears and triangular face

At the age of 25 months, growth was restricted (length: 83 cm, z − 1.42) and a further slowdown was observed at the age of 3.5 years (89 cm, z − 2.4; 10.9 kg, z − 2.6). At that age, relative macrocephaly was reported (OFC 48 cm, z − 1.45). The patient exhibited a triangular face, asymmetrical palpebral fissures, epicanthal fold on the left side, short philtrum (Fig. 2a), fifth finger clinodactyly on the left side and fourth digit crease. Despite the patient showed a phenotype reminiscent to SRS; clinical scoring according to the Netchine-Harbison clinical scoring system (NH-CSS) only revealed a score of 1 out of 6 (feeding difficulties and/or low BMI).

The parents were of normal height (maternal height: 165 cm; paternal height: 176 cm). The older brother was healthy and of normal height; a younger brother, born in the year of presentation of the patient, thrived well.

By first line molecular SRS testing using methylation-specific multiplex ligation probe dependent amplification (MS MLPA) the characteristic molecular alterations on chromosomes 11p15, 7 and 14q32 were excluded. Molecular karyotyping (CytoScan™ HD Array, Life Technologies, Carlsbad/USA) revealed heterozygosity for a 2 Mb deletion in 1q21.1q21.2 (arr[GrCh37] 1q21.1q21.2(145,895,747_147,897,962) × 1) (Fig. 1a), as well as a 575 kb duplication in 10q11.21 (arr[GRCh37] 10q11.21(42,709,645_43,284,257) × 3). Whereas the 1q21q21.2 deletion region harbors more than 10 protein-coding genes and has already been reported to be pathogenic, there is no evidence that the gain in 10q11.21 has an impact on the phenotype. Both parents underwent banding cytogenetic analysis and fluorescence in situ hybridization (FISH) with probes containing the deleted region. Both parents had normal karyotypes without any signs of rearrangements. Deletions in 1q21.1q21.2 were reported in individuals several times previously, and nearly all of these individuals were clinically suspected to have SRS (Table 1a).

### Case 2

The male patient with a 77 kb deletion in 8q12.1 was born as the first child of healthy non-consanguineous Hungarian parents (paternal height: 168 cm, maternal height: 154 cm). One miscarriage in the 10th week of gestation has been experienced. The pregnancy resulted from spontaneous conception and was complicated by maternal cholestasis, GBS positivity (Group B Streptococcus infection) and IUGR. Abnormal color Doppler flowmetry prompted an urgent Caesarean section at 30 weeks of gestation. Birth weight, height and OFC were reduced (990 g (z − 1.79), 37 cm (z − 1.39), 27 cm (z -− .23)). Apgar scores were 8 and 9, respectively; the newborn required non-invasive ventilation in the first 4 days of his life. At the age of 10 days, his condition worsened due to sepsis, for which he required mechanical ventilation for three days, one unit of blood transfusion and eight days of combined antibiotic therapy. Echocardiogram and cranial US were normal. Hearing and vision are reported normal, development was in the normal range. At the age of 1 7/12 years growth was restricted (80 cm, z -1.45), weight was 7.1 kg (z -3.87). Microcephaly was documented as well (44 cm, z -4.06). The patient exhibited a triangular face and a protruding forehead (Fig. 2b). He developed feeding difficulties requiring a nasogastric tube for two years. Asymmetry was not reported. By applying the NH-CSS, a score of 2 out of 6 was obtained.

Molecular testing for the SRS typical (epi)mutations on chromosomes 11p15, 7 and 14q32 by MS MLPA was negative. By molecular karyotyping (CytoScan™ HD Array), a 77 kb deletion affecting the *PLAG1* and the *CHCHD7* gene was identified (arr[GRCh37] 8q12.1(57,079,399_57,155,945) × 1); de-novo occurrence was proven by quantitative PCR of the parental DNA samples. In comparison to the other described cases carrying *PLAG1* deletions, this patient exhibited the smallest deletion reported up to now (Table 1b).

## Discussion

Both the phenotypic and molecular heterogeneity is a challenge for the clinical and genetic diagnosis of patients with SRS features. In fact, the recently consented Netchine-Harbison clinical scoring system [1] is a valuable tool to clinically diagnose SRS patients in which the characteristic molecular alterations in chromosomes 11p15 and 7 have been ruled out. However, even in these molecular subgroups the phenotypic range is broad and a considerable number of chromosome 11p15 and 7 patients do not fulfil the NH-CSS criteria. The same heterogeneity becomes obvious also for patients with mutations in the genes, which have been identified to cause SRS, i.e. *IGF2, PLAG1*, and *HMGA2* (for review: [4, 13, 15, 16]).

Accordingly, the contribution of different genomic loci to the SRS phenotype is reflected by the suggestion of five different OMIM entries: chromosome 11p15.5 (IC1: SRS1, #180,860; *IGF2*: SRS3, #616,489), 7p13-q32 (SRS2, #618,905), 8q12.1 (SRS4, *PLAG1*, #618,907), and 12q14 (SRS5, *HMGA2*, #618,908).

Though an OMIM entry has not been assigned yet for 1q21 deletions as another SRS region, the identification of the seventh patient with SRS features, including the key feature relative macrocephaly (Table 1a, Fig. 1a), reveals the relevance of this chromosomal region in the etiology of the disease. It should be noted that this region is not identical but distal to the already established 1q21 microdeletion syndrome (OMIM #612,474). The smallest critical region in all patients comprises 1.17 Mb (hg19: 146,641,600–147,735,011) and includes several genes, but none is known to be associated with altered growth. However, haploinsufficiency of *BCL9* and *CHD1L* have been discussed as candidate genes in 1q21 [10]. From a clinical point of view, the NH-CSS as the SRS clinical scoring system could only be applied in three cases, and two of them had a score of four out of six. However, none of the patients showed relative macrocephaly according to the NH-CSS, and in the total cohort, one patient fulfilled this feature. However, in our patient with 1q21 deletion a relative macrocephaly was obvious, but did not fit in the defined range of the NH-CSS. Nevertheless, the evidence for distal 1q21 as SRS candidate region is strengthening and the annotation as a sixth SRS OMIM entry should be considered.

The OMIM entry #618,907 for 8q12.1 is based on patients with single base pair substitutions in *PLAG1* (for review: [13]), but meanwhile two families with > 2 Mb deletions including *PLAG1* and more than 30 genes have been reported [12] (Table 1b, Fig. 1b). We now report on a third case with a deletion, but this variant is much smaller (77 kb), and affects *PLAG1* as the only protein-encoding gene with known physiological function in this region. Clinically, 60% of patients with *PLAG1* alterations (CNVs and SNVs) fulfilled the NH-CSS clinical score, but it should be noted that relative macrocephaly as an SRS key feature with its current definition (head circumference ≥ 1.5 SD above birth weight and/or length) could only be documented in two out of five patients. This case finally confirms that *PLAG1* is the SRS causing gene in 8q12.1 and it can be assumed that the first deletion in this region reported in SRS in 1994 [14] comprised this gene as well.

Both here discussed molecular subcohorts of patients with SRS features illustrate the clinical heterogeneity of SRS and the difficulty to establish the clinical diagnosis of SRS based on the current definition of the NH-CSS. For a more precise clinical diagnosis, the application of Human Phenotype Ontology (HPO) terms has to be discussed. Particularly with respect to the definition of the feature “relative macrocephaly” an expansion of the range needs to be considered. Furthermore, in daily clinical diagnostic routine, numerous patients are diagnosed as SRS due to their growth pattern and facial appearance, but do not pass the NH-CSS scoring as SRS. Since not even all of the patients with the typical SRS changes in 11p15 always fulfil the clinical score, it should be discussed to use the term SRS spectrum to reflect the clinical heterogeneity of the disorder.

In both cases reported here, the molecular diagnoses were established by array-based molecular karyotyping. This confirms that CNV analysis is an essential tool in the diagnostic workup of patients with SRS features and has to be included in the diagnostic algorithm [1].

In the future, it will be interesting to see whether the increasing implementation of improved diagnostic NGS-based approaches will replace the separate CNV analysis by array analysis as WES- or WGS-assays also allow CNV detection [7]. These NGS-assays have the advantage of covering nearly all genes with a high resolution whereas several commercially available routine diagnostic arrays do not cover all clinically relevant CNVs. An example is the *GH1* gene: In our diagnostic cohort of patients with growth retardation, we recently missed a homozygous *GH1* deletion by molecular karyotyping with the CytoScan™ HD Array as this assay does not cover this gene. However, we identified the alteration by WES (unpublished data). This example, as well as the broad pattern of molecular alterations identified in patients with SRS features, highlight the requirement of applying comprehensive assays, starting with first tier tests targeting aberrant methylation at different imprinted loci (e.g. on chromosomes 11, 7, 14, 16 and 20), followed by WES and WGS addressing both single nucleotide variants as well as CNVs [7]. Furthermore, the implementation of NGS-based approaches also covering the methylome and the transcriptome is conceivable. These tests would significantly increase the detection rate at a lower cost than today, but it is out of question that the application of these comprehensive tests significantly increases the risk/probability of identifying incidental findings. Like for genetic testing in general, the implementation of high-throughput tests in diagnostics of SRS and other imprinting disorders therefore needs a careful deliberation about the advantages and disadvantages for the patients and their families.

## Conclusions

The two reported cases with SRS features corroborate the molecular heterogeneity of the disease; also our second case confirms the *PLAG1* locus as SRS-disease-causing. In addition, the distal 1q21 region can be regarded as another SRS candidate region, and should therefore be included in the routine diagnostic workup of SRS.

## Methods

First line molecular testing for SRS comprised methylation-specific multiplex ligation probe dependent amplification (MS MLPA: assays ME030-C1, ME032-A1, ME034-C1, mrc Holland, Amsterdam/NL) to identify the characteristic molecular alterations on chromosomes 11p15, 7 and 14q32. Molecular karyotyping was performed by SNP array analysis (CytoScan™ HD Array, Life Technologies, Carlsbad/USA). All analyses were performed according to the manufacturers protocols.

## Data Availability

Not applicable.

## References

[1] Wakeling EL, Brioude F, Lokulo-Sodipe O, O'Connell SM, Salem J, Bliek J (2017). Diagnosis and management of Silver-Russell syndrome: first international consensus statement. Nat Rev Endocrinol.

[2] Eggermann T, Bruck J, Knopp C, Fekete G, Kratz C, Tasic V (2020). Need for a precise molecular diagnosis in Beckwith-Wiedemann and Silver-Russell syndrome: what has to be considered and why it is important. J Mol Med (Berl).

[3] Tumer Z, Lopez-Hernandez JA, Netchine I, Elbracht M, Gronskov K, Gede LB (2018). Structural and sequence variants in patients with Silver-Russell syndrome or similar features-curation of a disease database. Hum Mutat.

[4] Abi Habib W, Brioude F, Edouard T, Bennett JT, Lienhardt-Roussie A, Tixier F (2018). Genetic disruption of the oncogenic HMGA2-PLAG1-IGF2 pathway causes fetal growth restriction. Genet Med.

[5] Meyer R, Begemann M, Hubner CT, Dey D, Kuechler A, Elgizouli M (2021). One test for all: whole exome sequencing significantly improves the diagnostic yield in growth retarded patients referred for molecular testing for Silver-Russell syndrome. Orphanet J Rare Dis.

[6] Inoue T, Nakamura A, Iwahashi-Odano M, Tanase-Nakao K, Matsubara K, Nishioka J (2020). Contribution of gene mutations to Silver-Russell syndrome phenotype: multigene sequencing analysis in 92 etiology-unknown patients. Clin Epigenet.

[7] Alhendi ASN, Lim D, McKee S, McEntagart M, Tatton-Brown K, Temple IK, et al. Whole-genome analysis as a diagnostic tool for patients referred for diagnosis of Silver-Russell syndrome: a real-world study. J Med Genet. 2021. 10.1136/jmedgenet-2021-107699 34135092

[8] Spengler S, Begemann M, Ortiz Bruchle N, Baudis M, Denecke B, Kroisel PM (2012). Molecular karyotyping as a relevant diagnostic tool in children with growth retardation with Silver-Russell features. J Pediatr.

[9] Azzi S, Salem J, Thibaud N, Chantot-Bastaraud S, Lieber E, Netchine I (2015). A prospective study validating a clinical scoring system and demonstrating phenotypical-genotypical correlations in Silver-Russell syndrome. J Med Genet.

[10] Cottrell E, Cabrera CP, Ishida M, Chatterjee S, Greening J, Wright N (2020). Rare CNVs provide novel insights into the molecular basis of GH and IGF-1 insensitivity. Eur J Endocrinol.

[11] Vado Y, Pereda A, Llano-Rivas I, Gorria-Redondo N, Diez I, Perez de Nanclares G (2020). Novel variant in PLAG1 in a familial case with Silver-Russell syndrome suspicion. Genes (Basel)..

[12] Fernandez-Fructuoso JR, De la Torre-Sandoval C, Harbison MD, Chantot-Bastaraud S, Temple K, Lloreda-Garcia JM (2021). Silver Russell syndrome in a preterm girl with 8q12 1 deletion encompassing PLAG1. Clin Dysmorphol.

[13] Brereton RE, Nickerson SL, Woodward KJ, Edwards T, Sivamoorthy S, Ramos Vasques Walters F (2021). Further heterogeneity in Silver-Russell syndrome: PLAG1 deletion in association with a complex chromosomal rearrangement. Am J Med Genet A.

[14] Schinzel AA, Robinson WP, Binkert F, Fanconi A (1994). An interstitial deletion of proximal 8q (q11–q13) in a girl with Silver-Russell syndrome-like features. Clin Dysmorphol.

[15] Masunaga Y, Inoue T, Yamoto K, Fujisawa Y, Sato Y, Kawashima-Sonoyama Y (2020). IGF2 Mutations. J Clin Endocrinol Metab.

[16] Hubner CT, Meyer R, Kenawy A, Ambrozaityte L, Matuleviciene A, Kraft F (2020). HMGA2 variants in silver-russell syndrome: homozygous and heterozygous occurrence. J Clin Endocrinol Metab.

